# Charcot–Bouchard aneurysms revisited: clinicopathologic correlations

**DOI:** 10.1038/s41379-021-00847-1

**Published:** 2021-06-14

**Authors:** Shino Magaki, Zesheng Chen, Mohammad Haeri, Christopher K. Williams, Negar Khanlou, William H. Yong, Harry V. Vinters

**Affiliations:** 1 Section of Neuropathology, Department of Pathology and Laboratory Medicine, Ronald Reagan UCLA Medical Center and David Geffen School of Medicine, Los Angeles, CA USA; 2 Department of Neurology, Ronald Reagan UCLA Medical Center and David Geffen School of Medicine, Los Angeles, CA USA; 3 Brain Research Institute, Ronald Reagan UCLA Medical Center and David Geffen School of Medicine, Los Angeles, CA USA; 4 Present Address: Department of Pathology and Laboratory Medicine and Alzheimer Disease Research Center, University of Kansas Medical Center, Kansas City, KS USA; 5 Present Address: Department of Pathology and Laboratory Medicine, University of California—Irvine School of Medicine, Irvine, CA USA

**Keywords:** Brain, Cerebrovascular disorders

## Abstract

Intracerebral hemorrhage (ICH) is a significant cause of morbidity and mortality worldwide. Hypertension and cerebral amyloid angiopathy (CAA) are the most common causes of primary ICH, but the mechanism of hemorrhage in both conditions is unclear. Although fibrinoid necrosis and Charcot–Bouchard aneurysms (CBAs) have been postulated to underlie vessel rupture in ICH, the role and significance of CBAs in ICH has been controversial. First described as the source of bleeding in hypertensive hemorrhage, they are also one of the CAA-associated microangiopathies along with fibrinoid necrosis, fibrosis and “lumen within a lumen appearance.” We describe clinicopathologic findings of CBAs found in 12 patients out of over 2700 routine autopsies at a tertiary academic medical center. CBAs were rare and predominantly seen in elderly individuals, many of whom had multiple systemic and cerebrovascular comorbidities including hypertension, myocardial and cerebral infarcts, and CAA. Only one of the 12 subjects with CBAs had a large ICH, and the etiology underlying the hemorrhage was likely multifactorial. Two CBAs in the basal ganglia demonstrated associated microhemorrhages, while three demonstrated infarcts in the vicinity. CBAs may not be a significant cause of ICH but are a manifestation of severe cerebral small vessel disease including both hypertensive arteriopathy and CAA.

## Introduction

Intracerebral hemorrhage (ICH) accounts for ~10–15% of strokes in the West and 20–30% of strokes in Asia, with two million cases per year worldwide [1]. It is the stroke type associated with the highest mortality, with 1 month survival of 40% and significant morbidity with functional independence rate of around 10–40% [2, 3]. Although the incidence of ischemic strokes has decreased in recent decades, the incidence of ICH has remained stable [4]. In primary ICH, hypertension is thought to be the underlying cause in 65% of cases, followed by cerebral amyloid angiopathy (CAA). Secondary ICH is caused by various etiologies such as coagulopathy, berry/saccular aneurysms, vascular malformations, and tumors [3].

The most common location for ICH is the deep gray matter including basal ganglia and thalamus followed by the cerebral hemispheres, cerebellum and brainstem, predominantly the pons [1, 5]. Hypertensive hemorrhage frequently involves deep gray matter but can occur anywhere in the brain [6, 7]. CAA, characterized by the deposition of amyloid β (Aβ) preferentially in the walls of small- to medium-sized arteries and arterioles within the cortex and leptomeninges and less commonly in capillaries and veins, is associated solely with lobar hemorrhage due to the location of the involved vessels and has a higher risk for recurrence and poststroke dementia [6–8]. CAA is seen in the majority of patients with Alzheimer disease and in 20–40% of the nondemented elderly, with increasing prevalence with age in nondemented individuals [8–10]. Elevated blood pressure is associated with ICH recurrence regardless of location [4], and lowering blood pressure can decrease risk from hemorrhage in both hypertension and CAA-associated ICH [11].

Despite the well-known risk factors, the pathogenesis of ICH is unclear, and the site of bleeding has rarely been demonstrated histologically due to the difficulty in examining tissue destroyed by hemorrhage as well as secondary bleeding caused by the disruption of surrounding arteries [12, 13]. In hypertension, the cause of hemorrhage is thought to be elevated blood pressure-induced degenerative changes in the penetrating arterioles leading to rupture [14]. However, the precise nature of the degenerative changes is uncertain, although it has been attributed to fibrinoid necrosis, with deposition of plasma proteins including fibrin in the arteriolar wall with accompanying degeneration of smooth muscle cells, and Charcot–Bouchard aneurysms (CBAs) [5, 12, 15–17]. CBAs, also known as miliary aneurysms or microaneurysms, are small aneurysms that arise from arterioles usually less than 300 µm in diameter [18]. They were first described by Charcot and Bouchard in 1868 as a cause of hypertensive hemorrhage when they rupture [18, 19]. Since their description over 100 years ago, there has been controversy as to their very existence, prevalence and significance as a cause of ICH [15, 16].

Microaneurysms are also seen in CAA as one of the CAA-associated microangiopathies, which also include fibrinoid necrosis and “double barrel” or “lumen within a lumen” appearance, most often in severe CAA [10, 20]. The pathogenesis of hemorrhage in CAA is also not entirely clear but is thought to result from replacement of the smooth muscle cells of the media by amyloid with resultant weakening of the vessel walls and consequent rupture [10, 21, 22]. Fibrinoid necrosis and microaneurysms have also been associated with ICH in sporadic and familial CAA [10, 23–25]. Recent studies on microaneurysms are sparse and although previous studies have examined CBAs in the setting of hypertension or CAA generally separately, later studies, especially those investigating CAA, have shown that such a distinction may be artifactual [16, 26, 27]. Hypertension and CAA commonly co-exist [26, 28], and it has recently been proposed that hypertensive arteriopathy and CAA may be on a spectrum of age-related small vessel diseases (SVDs) with common underlying mechanisms including blood–brain barrier (BBB) dysfunction and impaired perivascular Aβ clearance [29, 30]. In this study, we describe the clinicopathologic features of microaneurysms encountered during routine brain autopsy in the setting of hypertension and/or CAA.

## Materials and methods

We searched the database of autopsies including brain or brain only autopsies, performed at UCLA Medical Center, a tertiary academic medical center, between 6/1/2002 and 6/1/2020 (inclusive) for CBAs. From a total of 2749 cases, 12 subjects with CBAs were identified with demographic and clinical information obtained from the medical records. Although we have a standard protocol in the examination and reporting of brain autopsies, and provide a high level of granularity in the neuropathology reports, our autopsies were initially assessed by different pathologists. Cases included in this study were re-reviewed by two pathologists for confirmation of CBAs. Because of the controversial nature of CBAs, partly due to their rarity on histologic examination and differing methods employed to study them, there is no consensus criteria for CBAs. However, in the literature they have been reported to occur in arterioles typically <300 µm in diameter, in cases where the parent arteriole is apparent [16, 18, 31], with the CBA itself ranging from 50 to 2500 µm [18, 32]. In this study, CBAs were defined as segmental dilatations, <2500 µm in diameter, in arterioles with or without luminal narrowing/occlusion by fibrosis or fibrin. CBAs in the leptomeningeal vessels are not well defined and have been rarely reported [18, 19, 33, 34], one lesion described as originating in the subarachnoid space and expanding as it extended into the cortex [18] and another shown in the setting of CAA [34]. Rare vascular lesions in the leptomeninges that were similar in appearance to CBAs in the parenchyma were categorized as leptomeningeal CBAs in this study. Because of the UCLA Dementia Brain Bank at Mary S. Easton Center for Alzheimer’s Disease Research, previously part of the Alzheimer’s Disease Research Centers, many of the autopsies had been performed on patients with dementia and 9 of our 12 subjects had such a history. For patients without a history of dementia, representative sections were taken from multiple areas of the cortex with subcortical white matter, basal ganglia, hippocampus, brainstem, and cerebellum and submitted for routine examination on formalin fixed paraffin embedded (FFPE) tissue sectioned at 4–6 µm in thickness and stained with hematoxylin and eosin (H&E). For patients with a history of dementia, brains were extensively sampled according to the UCLA dementia protocol including representative sections from the frontal, temporal, parietal, and occipital cortices, hippocampus, entorhinal cortex, amygdala, basal ganglia, brainstem, and cerebellum. In addition to H&E staining, immunohistochemistry was performed on select blocks using antibodies to β-amyloid 1-42 (1:150, EMD Millipore, rabbit polyclonal, AB5078P), β-amyloid 1-40 (1:400, EMD Millipore, rabbit polyclonal, AB5074P), phospho-tau (1:200, Thermo Fisher, mouse monoclonal, AT8), and alpha-synuclein (1:450, EMD Millipore, rabbit polyclonal, AB5038). Sections were incubated with the primary antibody followed by either horse anti-mouse or horse anti-rabbit secondary antibody conjugated to horseradish peroxidase (MP7402 and MP7401; Vector Laboratories, Burlingame, CA). Antibody reactivity was visualized with N’N Diaminobenzidine as chromogen (no. SK-4100; Vector Laboratories) and counterstained with hematoxylin.

For brains processed according to the dementia protocol, standard diagnostic criteria were used to assess neuropathologic substrates of dementia [35]. The overall severity of CAA was graded according to the Vonsattel criteria as none, mild, moderate, or severe [23] with the presence or absence of capillary CAA noted as recommended by recent guidelines in assessing CAA [36] and cerebrovascular pathology [37]. The severity of cerebrovascular disease, infarcts, hemorrhage, and presence of calcifications were also examined. Assessment of the degree of atherosclerotic arterial narrowing was based on gross and microscopic estimates of stenosis of major branches of the circle of Willis, including basilar and vertebral arteries, as none, mild: <20%, moderate: 20–50%, and severe: >50% [34]. Arteriolosclerosis was graded as none, mild, moderate, and severe based on the degree of thickening and fibrosis of the walls of arterioles [38]. Infarcts were classified as cystic or macroinfarcts (≥1 cm), lacunar infarcts (grossly visible but <1 cm), and microinfarcts (not visible grossly but detected in histological brain sections). Parenchymal hemorrhage was classified as large hemorrhage, visible grossly, or microhemorrhage, seen only on microscopic examination [34, 37].

Sections from FFPE blocks containing CBAs were additionally stained with Masson trichrome stain which stains collagen blue and fibrin red [39], β-amyloid 1-40 as above if not already done, CD163 (1:500, BioRad, mouse monoclonal, EDHu-1) and CD206 (1:50, Santa Cruz, mouse monoclonal, C-10) for perivascular macrophages, and PDGFR-β (1:50, Santa Cruz, mouse monoclonal, 18A2) for pericytes in a subset of the cases. As CBAs are small, not all CBAs were retained in deeper sections and thus not all stains could be performed on every case. The CBAs were examined for the following histologic features: Aβ deposition, hyalinization/fibrosis, fibrinoid necrosis, associated hemorrhage, perivascular hemosiderin (defined as hemosiderin in perivascular spaces as opposed to microhemorrhage in which hemosiderin is seen within brain parenchyma [37]), mural and perivascular macrophages on H&E, CD163 and/or CD206 immunostains, surrounding pericytes on PDGFR-β immunostain, and calcifications. The sections containing CBAs were also further separately graded for arteriolosclerosis and perivascular hemosiderin, according to the VCING criteria on a 0–3 scale: for arteriolosclerosis 0 = normal, 1 = mild fibrosis, 2 = moderate fibrosis, and 3 = severe fibrosis; and for perivascular hemosiderin 0 = absent, 1 = <3 hemosiderin granule deposits, 2 = 3–5, and 3 = >5 [37].

## Results

### Demographic and clinical data

The demographic and clinical characteristics, including neurologic diagnoses, of the 12 subjects with CBAs are summarized in Table 1. Most subjects were of advanced age with an average age of 80.9 ± 8.8 (standard deviation) and ranging from 61 to 93 years. The majority were Caucasian, two were Hispanic and one was African American. The subjects included nine males and three females. Nine subjects had dementia, most with Alzheimer disease. Detailed clinical history was unavailable in one subject who was transferred from an outside hospital with a large parenchymal hemorrhage and died soon after, except for reported history of cocaine use. The majority of subjects (8 of 12) had hypertension, two of whom also had severe coronary artery disease, one with type 2 diabetes mellitus complicated by peripheral arterial disease and end-stage renal disease and the other with history of myocardial infarction (MI).

**Table 1:** Demographic and clinical data for patients studied.

Case	Age	Sex	Race	Clinical characteristics	
				Medical history	Neurologic diagnoses
**1**	90	M	C	HTN, hypercholesterolemia, sick sinus syndrome status post pacemaker, multiple decubitus ulcers	Vascular dementia with multiple infarcts
**2**	93	F	C	HTN, tumor on chest of unknown etiology, osteoporosis	Alzheimer dementia with Parkinsonian features, minor depression, anxiety
**3**	75	M	C	Arrhythmia, hypothyroidism, recurrent pancreatitis	Alzheimer dementia, herpes zoster involving cranial nerves
**4**	79	M	C	HTN, Stanford type B aortic dissection/aneurysm, hernia	None
**5**	82	M	AA	Unknown other than history of cocaine use	Intracerebral hemorrhage
**6**	87	F	C	HTN, atrial fibrillation, lung cancer status post right lobectomy, COPD, CKD, hypothyroidism, osteoarthritis	Alzheimer dementia, multiple small infarcts, intermittent cranial nerve VI palsy
**7**	80	M	C	GERD, osteoarthritis	Dementia with Lewy bodies, small infarct, history of head injury, adult attention deficit disorder
**8**	89	M	C	Benign prostatic hyperplasia	Alzheimer dementia
**9**	61	M	H	HTN, CAD with stent thrombosis, DM2 with peripheral arterial disease, ESRD	None
**10**	78	M	C	HTN	Alzheimer dementia
**11**	84	F	C	HTN, hypothyroidism	Alzheimer dementia
**12**	73	M	H	HTN, hypercholesterolemia, CAD with myocardial infarction, COPD	Parkinson disease, vascular dementia with multiple strokes

*M* male, *F* female, *C* Caucasian, *AA* African American, *H* Hispanic, *HTN* hypertension, *CAD* coronary artery disease, *CKD* chronic kidney disease, *DM2* type 2 diabetes mellitus, *ESRD* end-stage renal disease, *COPD* chronic obstructive pulmonary disease.

### Pathologic findings

Eight subjects underwent complete autopsy examination while four cases had “brain only” examinations. The cause of death for the six subjects who had dementia and also underwent a full autopsy was pneumonia, consistent with prior studies showing that pneumonia is the most common cause of death in patients with dementia (Table 2) [40]. The two subjects with known clinical history who were cognitively intact died from cardiovascular etiologies, one from cardiogenic shock in the setting of severe systemic and coronary artery atherosclerosis leading to MI and the other from a dissecting descending aortic aneurysm with rupture. The one other subject with an aortic aneurysm demonstrated at autopsy had a history of Alzheimer dementia, hypertension and hypothyroidism as well as severe systemic and coronary artery atherosclerosis.

**Table 2:** Pathologic findings at autopsy.

Case	Cause of death	Significant non-CNS pathologic findings		Neuropathologic findings								
			Neurodegen	Cerebrovascular disease						Charcot–Bouchard aneurysms		
				CAA^a^ (art)	CAA (cap)	Arterio	Athero	Infarcts (region)	Hem	Focality	Anatomic region	Location
1	PNA	sev SA, mod CAD, PNA	Early AD change (NFT B/B III, mod DP, rare NP)	mod	−	sev	mod	Lacunar infarcts (BG), microinfarcts (parietal, occipital)	None	Single	Frontal	Leptomeninges
2	Brain only	n/a	AD change (NFT B/B VI, abundant DP, scattered NP)	sev	+	sev	mod	Microinfarcts (prefrontal, temporal, HP, BG, CBL)	None	Multifocal	Occipital, basal ganglia	Leptomeninges, putamen
3	PNA	mod SA, aspiration PNA, arteriolar nephrosclerosis, renal adenoma, prostate CA	AD change (NFT B/B VI, abundant DP, frequent NP)	sev	+	mod	mild	None	None	Multifocal	Temporal, occipital, frontal	Superficial and mid cortex, leptomeninges
4	Thoracic aortic dissection	Ruptured descending thoracic AA, heart with LVH, mod SA and CAD	None	None	−	mod to sev	mod	None	None	Multifocal	Basal ganglia	Caudate, internal capsule
5	ICH	n/a	None	mod	+	mod	mod	Microinfarcts (midbrain)	Large hem (frontal, basal ganglia)	Multifocal	Occipital	Superficial and mid cortex
6	Brain only	n/a	AD change A3B3C3	mild	+	mod	mild to mod	None	Microhem around CBA	Single	Basal ganglia	Putamen
7	Brain only	n/a	AD change A3B3C3, DLBD	sev	+	mod to sev	sev	Microinfarcts (cerebral and cerebellar hemispheres)	None	Single	Frontal	Superficial cortex
8	PNA	mod SA and CAD, aspiration PNA, hemorrhagic gastritis	AD change A3B3C3, DLBD	sev	+	mod	sev	Microinfarcts (frontal)	None	Single	Occipital	Superficial cortex
9	Cardiogenic shock	sev SA and CAD, MI	None	None	−	Mild to mod	Mild to mod	Lacunar infarcts with secondary hem (CBL)	None	Single	Temporal	Mid cortex
10	PNA	sev SA, mod CAD, aspiration PNA, mild to mod arterial and arteriolar nephrosclerosis	mild AD change A1B2C0	None	−	mod to sev	mild to mod	None	Microhem around CBA	Single	Basal ganglia	Putamen
11	PNA	sev SA and CAD, MI, LVH, abdominal AA, pulmonary hypertension with RVH, PNA, right renal artery stenosis, bilateral nephrosclerosis	AD change A3B3C3	mild	−	sev	sev	None	None	Multifocal	Basal ganglia	Putamen, globus pallidus
12	PNA	sev CAD, MI, PNA, small jejunal GIST, chronic pyelonephritis	Progressive supranuclear palsy, AD change A3B3C3	mild	−	sev	sev	Macroinfarcts (frontal, occipital, parietal, BG), microinfarcts (HP, amygdala)	None	Single	Occipital	Gray-white matter junction

*n/a* not applicable as brain only autopsy, *arterio* arteriolosclerosis, *athero* atherosclerosis of circle of Willis, *AD* Alzheimer disease, *AA* aortic aneurysm, *art* arteriolar, *B/B* Braak and Braak stage, *BG* basal ganglia, *CA* carcinoma, *CAD* coronary artery disease, *cap* capillary, *CAA* cerebral amyloid angiopathy, *CBA* Charcot–Bouchard aneurysm, *CBL* cerebellum, *DLBD* diffuse Lewy body disease, *DP* diffuse plaques, *GIST* gastrointestinal stromal tumor, *hem* hemorrhage, *HP* hippocampus, *ICH* intracerebral hemorrhage, *LVH* left ventricular hypertrophy, *MI* myocardial infarction, *microhem* microhemorrhage, *mod* moderate, *neurodegen* neurodegenerative disease, *NFT* neurofibrillary tangles, *NP* neuritic plaques, *PNA* pneumonia, *RVH* right ventricular hypertrophy, *SA* systemic atherosclerosis, *sev* severe, *+* present, *−* absent.

^a^Vonsattel grading.

On brain examination, all subjects had some degree of atherosclerosis and arteriolosclerosis with seven demonstrating infarcts, all multifocal, in the cortices, hippocampus, amygdala, basal ganglia, cerebellum, and/or midbrain. Nine subjects demonstrated CAA, six with both arteriolar and capillary CAA. The patient who was admitted for ICH showed a large hematoma involving the right basal ganglia and right frontal and temporal lobes with extension into the lateral, third and fourth ventricles, causing ventricular dilatation. Small amounts of blood were also seen in the subarachnoid space. There was moderate arteriolar and capillary CAA as well as microinfarcts in the midbrain, arteriolosclerosis with scattered vessels showing hyalinization, and moderate atherosclerosis with microatheromas in the vessels of the cerebellar white matter. CT angiogram did not show evidence for an intracranial aneurysm or stenosis/occlusion. The site of hemorrhage could not be determined, but the etiology is likely multifactorial given the history of cocaine use which is also associated with ICH [41]. The nine subjects who had a clinical history of dementia demonstrated AD neuropathologic change ranging from Braak and Braak stage III to VI and rare to frequent neuritic plaques. Two cases additionally had diffuse Lewy body disease. One patient who was clinically thought to have Parkinson disease and vascular dementia demonstrated on neuropathologic examination not only severe AD pathologic change and severe vascular disease with multiple infarcts but also features of progressive supranuclear palsy. A subject with clinical history of hypertension and hypercholesterolemia who was suspected to have vascular dementia, showed severe hyaline arteriolosclerosis with marked white matter rarefaction and multiple lacunar infarcts in the basal ganglia as well as cortical microinfarcts. He also demonstrated mild to moderate CAA. AD neuropathologic change was only mild.

### Characterization of Charcot–Bouchard aneurysms

**Fig. 1:**
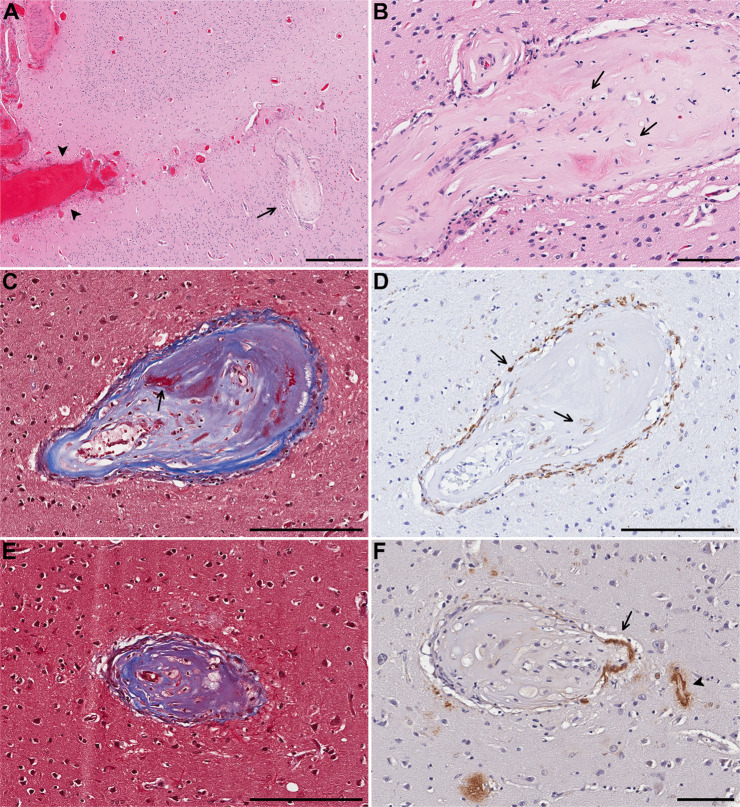
Case 5. **A** Charcot–Bouchard aneurysm (CBA) (arrow) in the superficial cortical layer and acute blood in subarachnoid space (arrowheads) and **B** higher magnification of the same CBA with fibrosis and foamy macrophages (arrows). **C** In a CBA in the mid cortex, trichrome stains collagen blue and focal fibrin red (arrow). **D** CD163 immunostain highlights macrophages surrounding and within the CBA (arrows). **E** Trichrome stain on a different CBA in the mid cortex also demonstrates fibrosis and macrophages. **F** Aβ40 immunostain highlights β-amyloid in the wall of the parent artery (arrow) and adjacent capillary (arrowhead). Scale bars: **A** = 500 µm, **B**, **F** = 100 µm, **C**–**E**: 200 µm).

Six subjects had one or more CBAs in the cortex, the most common anatomic location in which CBAs were detected in our cohort, followed by the basal ganglia, and leptomeninges (Table 3). Five subjects had CBAs in the basal ganglia. In nearly half of the subjects there was more than one CBA either in the same anatomic region or elsewhere. In the occipital cortex of the only subject (subject 5) with a large hemorrhage, multiple CBAs were identified that showed fibrosis with focal fibrinoid necrosis and foamy macrophages in their walls as well as one CBA in which amyloid deposition was seen in the “parent” vessel (Fig. 1). There was moderate leptomeningeal and cortical CAA with capillary involvement surrounding the CBAs.

**Table 3:** Histologic features of Charcot–Bouchard aneurysms (CBAs).

Feature associated with CBA	Number of cases (%)
Multifocality	5 (42%)
Location	
Cortex	6 (50%)
Leptomeninges	3 (25%)
Basal ganglia	5 (42%)
Hyalinization/fibrosis	10 (83%)
Fibrinoid necrosis	1 (8%)
Large hemorrhage	0 (0%)
Microhemorrhage	2 (17%)
Perivascular hemosiderin	2 (17%)
Perivascular macrophages (HE, CD163 and/or CD206)	10 (83%)
Macrophages within wall or fibrosis (HE, CD163)	6 (50%)
Pericytes (PDGFRβ)	0 out of 7^b^
Calcifications	2 (17%)
CAA^a^	3 out of 5^b^

Features of background brain and other vessels	
Infarct(s) in same section as CBA	3 (25%)
Infarct(s) anywhere in brain	7 (58%)
Large hemorrhage or microhemorrhage in same section as CBA	0 (0%)
Large hemorrhage or microhemorrhage anywhere in brain	4 (33%)
Perivascular hemosiderin score in same section as CBA^c^	
≥3	8 (67%)
<3	3 (25%)
Arteriolosclerosis score in same section as CBA^c^	
≥3	3 (25%)
<3	9 (75%)
Atherosclerosis score	
Severe	5 (42%)
Mild to moderate	7 (58%)
CAA in same section as CBA^a^	6 out of 7
CAA anywhere in brain	9 (75%)
Moderate to severe	6 (50%)
Mild	3 (25%)
Capillary CAA present	6 (50%)
Calcification in same section as CBA	2 (17%)
Calcification anywhere in brain	3 (25%)

^a^Cortical/leptomeningeal locations only, in subjects with CAA.

^b^Out of cases in which CBAs still present in the deeper stained sections.

^c^Scoring according to the VCING criteria [37].

**Fig. 2:**
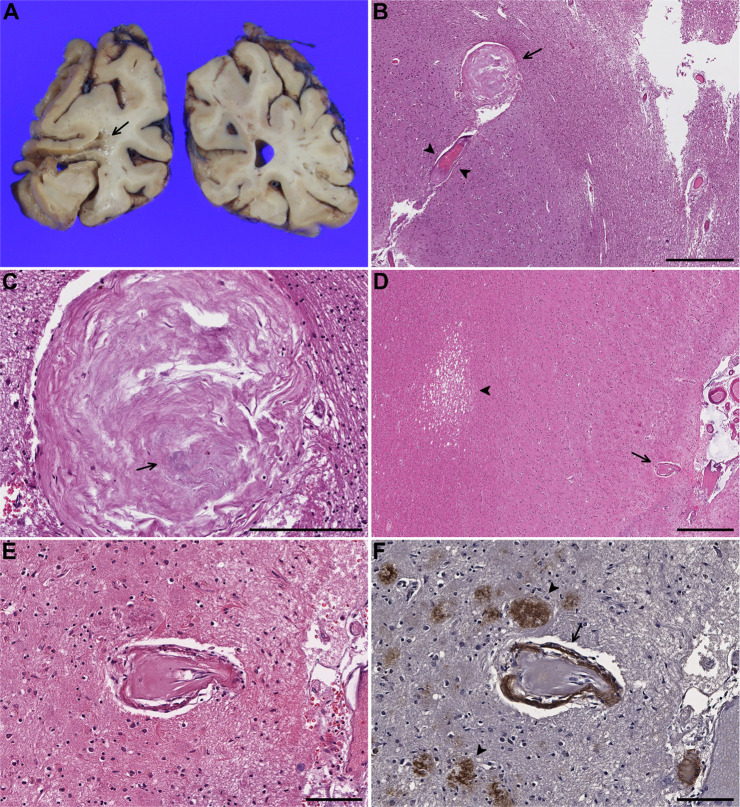
Case 12. **A** Cystic infarct in the left occipital lobe (arrow). **B** Microscopic examination shows an adjacent Charcot–Bouchard aneurysm (CBA) forming a fibrous ball (arrow) in close proximity to an arteriole with adventitial fibrosis (arrowheads). **C** Magnified view of the structure highlighted by an arrow in **B** demonstrates punctate calcifications (arrow). **Case 7.** **D** CBA (arrow) in proximity to a microinfarct (arrowhead) in the subcortical white matter. **E** Magnified view of the CBA (**D**) shows hyalinization and splitting of the vessel wall with **F** extensive amyloid deposition (arrow) and scattered senile plaques in the vicinity (arrowheads) highlighted by Aβ40 immunostain. Scale bars: **B** = 600 µm, **C** = 200 µm, **D** = 500 µm, **E**, **F** = 100 µm.

In subjects with cortical and leptomeningeal CBAs, amyloid deposition was seen in the CBAs or “parent” vessels in three of five cases in which CBAs were still present in deeper Aβ40 immunostained sections. Six out of seven cases had CAA-affected vessels in the same section as the CBA whether or not the CBA itself or “parent” vessel showed amyloid deposits. All of the CBAs in the cortex were seen in the mid to superficial cortical layers except in one subject (subject 12) who had a single CBA at the gray-white matter junction forming a “fibrous ball/nodule,” thought to represent a CBA with contents that had undergone organization [16, 18, 42], with focal microcalcifications in the CBA, seen adjacent to a cystic infarct (Fig. 2A–C). Although the CBA was no longer seen in deeper sections immunostained for Aβ40, CAA-affected vessels were present in the vicinity. One subject showed a cortical CAA-associated CBA adjacent to a microinfarct in the white matter (Fig. 2D–F), one of many microinfarcts in both white matter and cortex in the section, while another subject had a leptomeningeal CAA-associated CBA near a cortical microinfarct.

**Fig. 3:**
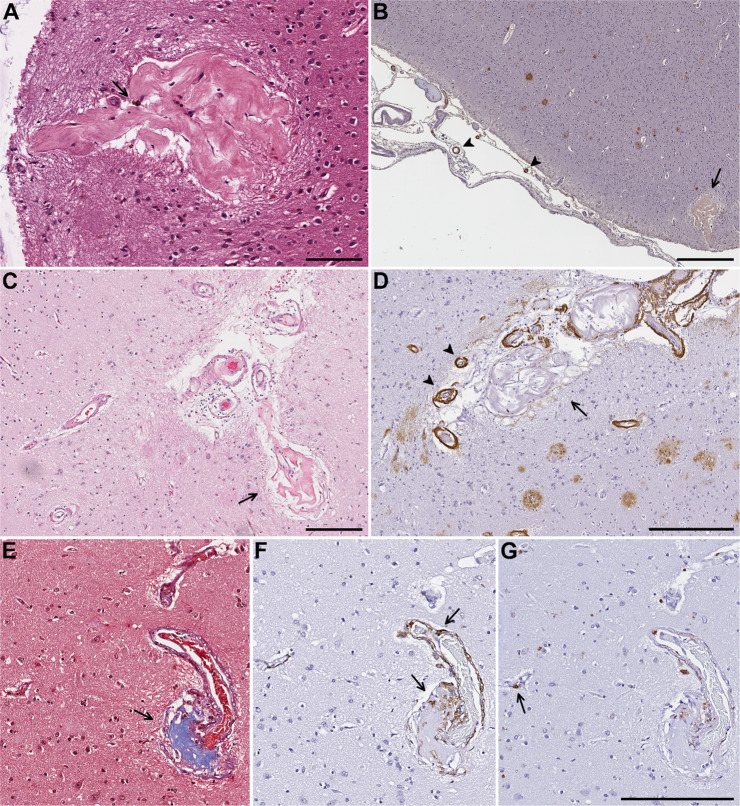
Case 8. **A** CBA forming a fibrous ball with rare hemosiderin deposits (arrow). **B** On Aβ40 immunohistochemistry the same CBA (arrow) is in proximity to CAA-affected leptomeningeal vessels (arrowheads). **Case 3.** **C** Superficial cortical and leptomeningeal CBAs forming a fibrous ball (arrow) on H&E and Aβ40 immunostain (**D**) which shows amyloid deposition in the residual walls of the CBA as well as severe CAA in adjacent vessels with a “double barrel” lumen (arrowheads). **E** Trichrome stain demonstrates fibrosis in a CBA (arrow) arising from the parent artery superiorly. **F** CD163 immunohistochemistry highlights perivascular macrophages and macrophages within the fibrosis (arrows). **G** PDGFR-β immunostain labels a pericyte with “bump-on-a-log” morphology (arrow) in an adjacent capillary with no definite pericyte-like cells identified around the CBA. Scale bars: **A** = 100 µm, **B** = 500 µm, **C** = 200 µm, **D** = 300 µm, **G** = 200 µm.

The majority of our cases (10 of 12) showed fibrosis or hyalinization with several forming fibrous balls. Calcifications were seen in two CBAs. In one superficial cortical CBA forming a fibrous ball with rare hemosiderin deposits, there were CAA-affected leptomeningeal vessels in proximity (Fig. 3). Ten subjects demonstrated perivascular macrophages or macrophages within the vessel wall or fibrous connective tissue of the CBA seen either on H&E, CD163 and/or CD206 immunohistochemistry. No pericytes were seen in seven CBAs that persisted in deeper sections immunostained with PDGFR-β, but were present in surrounding smaller vessels in all cases, consistent with CBAs typically arising from small penetrating arteries and parenchymal arterioles whereas pericytes are seen in precapillary arterioles, capillaries, and postcapillary venules [43, 44]. PDGFR-β and PDGF-B knock out mice have leaky and tortuous vessels with formation of microaneurysms, mainly in capillaries, and die at birth due to hemorrhage and edema [43, 45]. There is increasing evidence that pericytes are critical in the formation and maintenance of the BBB, regulation of cerebral blood flow and angiogenesis [44, 46]. Pericyte dysfunction leads to leakiness in the BBB, and lack of pericytes results in alterations in vessel architecture such as dilation of small and large vessels and formation of microaneurysms, similar morphologically to those seen in diabetic microangiopathy, raising speculation about possible associations with other vascular lesions such as CBAs [44, 45].

**Fig. 4:**
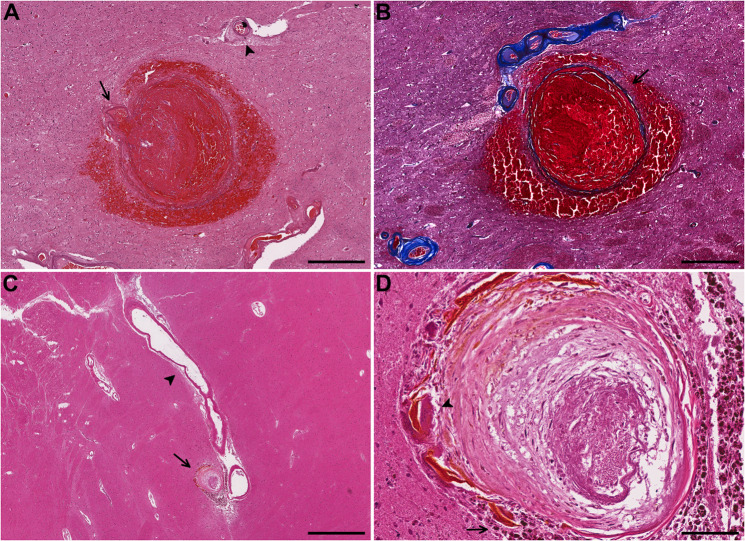
Case 6. **A** Acute hemorrhage surrounding a CBA in the putamen with parent arteriole on left (arrow) and adjacent arteriole showing arteriolosclerosis with adventitial fibrosis (arrowhead). **B** Trichrome stain on an adjacent section highlights thinning and splitting of the collagenous wall (arrow) demarcating it from the surrounding hemorrhage. **Case 10.** **C** CBA with surrounding old hemorrhage (arrow), likely arising from a branch of the lenticulostriate artery with arteriosclerosis (arrowhead) in the putamen. **D** Higher magnification showing surrounding hemosiderin laden macrophages (arrow) and multinucleated giant cells (arrowhead). Scale bars: **A**, **B** = 500 µm, **C** = 1000 µm, **D** = 100 µm.

CBA associated microhemorrhages were seen in two cases, both in the basal ganglia, one acute with red blood cells in the surrounding brain parenchyma and the other chronic with many hemosiderin laden macrophages, multinucleated giant cells, and hematoidin pigment (Fig. 4). Two CBAs showed only perivascular hemosiderin. Almost all cases had adjacent vessels showing perivascular hemosiderin, and all subjects showed arteriolosclerosis although the majority (75%) were mild to moderate in severity.

## Discussion

Charcot and Bouchard examined cases of ICH in which they separated blood vessels from regions of hemorrhage rinsed under water and found numerous structures attached to vessels that they described as miliary aneurysms, leading to the hypothesis that these aneurysms, now known as CBAs, were the cause of ICHs [18, 19, 31, 33]. Initially several investigators thought that these CBAs were not true aneurysms but rather false aneurysms resulting from extravascular hemorrhage and dissection of blood between the media and adventitia [47–49]. Subsequent studies, however, histologically confirmed the existence of CBAs, some at or near sites of hemorrhage or ischemic change [18, 49, 50].

In 1930, Green extensively sampled and performed serial sections on autopsy brains from ten patients with hypertension, atherosclerosis, and arteriosclerosis, including three with ICH, and found three microaneurysms in two patients [49]. The first patient had died from a hemorrhage likely arising from the choroid plexus in the fourth ventricle although the ruptured vessel could not be identified. However, two smaller hemorrhages each associated with a CBA were found in the pons and left frontal cortex with a very thin fibrous ring around an organizing blood-clot, unlikely to simply represent perivascular hemorrhage. The second patient demonstrated a thrombosed CBA associated with ischemic change in the pons [49].

Fisher examined the basal ganglia, pons, and cerebral cortex in 20 patients with history of hypertension, multiple small infarcts, and/or massive cerebral hemorrhage and severe atherosclerosis and found three types of miliary aneurysms—saccular, asymmetric fusiform, and lipohyalinotic—commenting that all three may have a common origin and represent the morphologic spectrum of CBAs [18]. He also described pseudoaneurysms, also called “bleeding or fibrin globes”, comprised of small ball hemorrhages with red blood cells and concentric rings of fibrin adherent to vessels that may have mimicked true aneurysms [18]. Saccular microaneurysms were described as “narrow-mouthed out-pouchings,” arising from small penetrating arteries that were found only in the deep gray matter and pons [18]. Lipohyalinotic microaneurysms showed fibrin deposition or fibrosis that sometimes formed a “fibrous ball,” frequently with foamy macrophages in the fibrosis leading to the term “lipohyalinosis.” These had a predilection for the superficial half of the cerebral cortex and were associated more with lacunar infarcts and small hemorrhages rather than massive ICH [51, 52]. He found only two examples of asymmetric fusiform microaneurysms that were seen deep in the cerebral cortex and thought to have caused lacunar infarcts in the vicinity [18]. Several decades later he described what may have been the first histologic demonstration of the site of bleeding in a case of hypertensive ICH, a ruptured microaneurysm that did not fit into any of the types of microaneurysms previously described [12]. In our cohort, no CBAs were seen in the vicinity of the hemorrhage in the one subject with ICH, but three subjects showed cystic or microinfarcts adjacent to CBAs, one forming a fibrous ball and one associated with CAA.

Some have described lipohyalinosis, with fibrosis and mural foamy macrophages, as the healed or chronic phase of fibrinoid necrosis [17], while others have argued that lipohyalinosis is synonymous to fibrinoid necrosis and does not result from fibrinoid necrosis [12, 16]. Fisher appears to have used lipohyalinosis interchangeably with fibrinoid necrosis although he described lipohyalinosis as a process, perhaps referring to a spectrum of histologic findings [12, 18]. Due to the confusion as to what the term actually describes and its misuse in describing various degenerative changes in small vessels, its usage has been discouraged [17]. It is agreed that lipohyalinosis is distinct from hyaline wall thickening seen in aging and arteriolosclerosis, for which hypertension is also a risk factor, despite the inclusion of “hyaline” in the name [17, 18, 53]. It is also different from the atheromas in larger arteries (microatheromas in smaller arteries and arterioles) of atherosclerosis despite the common finding of macrophages in the vessel wall [53]. Prominent vascular wall macrophages have also been described in amyloid-laden vessels in familial CAA, demonstrating that the changes of lipohyalinosis traditionally attributed to hypertensive arteriopathy can also be seen in CAA-affected vessels [25].

The frequency of CBAs detected in ICHs has ranged from rare to common, likely at least in part due to the difficulty in examining hemorrhagic tissue and the different methodologies employed. Ultrastructural examination of arteries collected from 11 autopsy brains of patients who died from hypertensive ICH and 20 lenticulostriate arteries from evacuated hematoma specimens in those undergoing surgery for ICH, identified 48 ruptured arteries, 2 of which were ruptured CBAs (5 unruptured CBAs were seen in 2 cases) [54]. The remainder of the arteries ruptured at or near arterial bifurcation points and showed arteriosclerosis and smooth muscle degeneration of the media [54]. Hinton et al. found tumors, CAA, hypertensive vascular changes, and one case of acute bacterial abscess, but no CBAs in 54 out of 84 surgical evacuation specimens which contained both blood and brain tissue from spontaneous intracerebral and intracerebellar hemorrhages in which the etiology was unknown preoperatively [55]. A later study evaluated surgical specimens obtained from meticulous evacuation of spontaneous ICHs in 29 consecutive patients with negative angiography using a surgical microscope followed by serial sectioning, and detected 11 CBAs [27]. This study also evaluated amyloid deposition using Congo red staining which was negative in the CBAs and parent arteries; however, microaneurysms and pseudoaneurysms were both counted as “microaneurysms” [27]. Fisher and Rosenblum each reported a case of ICH in which a ruptured CBA was the site of hemorrhage with both showing fibrinoid necrosis/lipohyalinosis at or adjacent to the site of rupture [12, 13].

In comparison to investigators who used serial sectioning in autopsy or surgical specimens, studies in which cerebral vessels were examined radiographically, by injecting a barium sulfate and gelatin mixture into the arteries and subsequently X-raying brain slices, have detected numerous microaneurysms [31, 32, 56]. Russell found CBAs in 14 out of 16 hypertensive (with one demonstrating poor filling by barium) and 10 out of 35 normotensive subjects (three with poor filling) while Cole and Yates found CBAs in 46 out of 100 hypertensive and 7 of 100 normotensive subjects [31, 57]. A later study that examined brain sections with high resolution microradiography after staining the endothelium with alkaline phosphatase found no CBAs in 35 hypertensive and 20 normotensive subjects and suggested that previous investigators may have mistaken tortuous vascular profiles for CBAs [56]. Moreover, assessment of microscopic CBAs may have been difficult with the limited resolution of plain radiographs. In the same study, the authors also examined 2800 routine autopsies and detected five CBAs, none associated with ICH and all over 70 years of age with advanced arteriosclerosis. Presence or absence of CAA was not addressed [56].

Similarly, our study of over 2700 routine autopsies also demonstrated few subjects with CBAs. All were above 70 years of age except for one subject, a 61-year-old male who had severe systemic and coronary artery atherosclerosis with MI and died from cardiogenic shock. As CAA rarely involves the basal ganglia, the basal ganglia CBAs in five subjects, all with history of hypertension, are likely hypertensive in etiology whether or not CAA was also present [26]. However, co-occurrence of hypertensive arteriopathy and CAA is common in this age group, and one subject with both cortical and basal ganglia CBAs had severe CAA as well as severe arteriolosclerosis [28]. The majority of cortical CBAs in our study were located in the superficial to mid cortex, similar to prior studies utilizing serial sections that have also found that lobar CBAs, including those associated with CAA, are commonly seen in the superficial cortical layers [18, 24]. This is in contrast to studies injecting contrast material into vessels that have found frequent aneurysms in the centrum semiovale as well as the cortex, although these studies examined select CBAs histologically as well [31, 57].

Miliary aneurysms are also seen in the context of CAA, classified as one of the CAA-associated microangiopathies (vasculopathies), which additionally include fibrinoid necrosis, “lumen within a lumen” appearance, inflammation, vascular fibrosis/scarring, and rarely calcifications, and are seen more often in severe CAA [10, 20]. Although bleeding in CAA-associated ICH has been attributed to vessel wall damage and fragility due to amyloid deposition, the mechanism is not fully understood, as the most common location of CAA-related ICH, fronto-parietal, does not entirely correspond with the area most severely affected by CAA, parieto-occipital [28, 58]. Furthermore, microhemorrhages seen on histologic examination do not correlate with vascular amyloid burden [6, 21]. In assessing microhemorrhages in CAA, van Veluw et al. showed that only one of seven microhemorrhages in which the involved vessel could be identified showed vascular Aβ deposition at the rupture site, and the density of amyloid-laden vessels was lower in the vicinity of microhemorrhages [21]. A different group demonstrated that lobar microhemorrhages were more frequent in parietal and frontal lobes and seen mainly in the white matter, while CAA was seen in pial and superficial cortical vessels [6]. Microinfarcts have been shown to correlate with severity of CAA both locally and globally, however [59, 60]. Similar to hypertensive ICHs, fibrinoid necrosis and microaneurysm formation have also been implicated in CAA-related hemorrhage [10, 20, 23, 61].

In a study of 25 autopsy cases of CAA, five subjects showed CAA-associated microangiopathies including aneurysmal vessels and fibrinoid necrosis, of whom all five had moderate to severe CAA and infarcts, and three had hemorrhages including two with massive ICH [20]. Vonsattel et al. compared 15 autopsy brains and two brain biopsies from patients with CAA-related ICH to 136 brains from patients without hemorrhage, approximately half of whom had CAA, and found that the histopathologic findings most consistently associated with hemorrhage in patients with CAA were a severe degree of CAA and fibrinoid necrosis, whether or not associated with microaneurysms [23]. Fibrinoid necrosis was seen only in patients with hemorrhage (12 of 17 subjects) and microaneurysms only in those with severe CAA [23]. In our cohort, fibrinoid necrosis was also only seen in the subject with ICH. In two-thirds of subjects with CAA, the degree of CAA was moderate to severe, including three without a history of hypertension who all had severe CAA with CBAs limited to the cortex and leptomeninges. Among the three subjects with mild CAA, all of whom had a history of hypertension, two had CBAs only in the basal ganglia, and one had a single CBA at the cortical-white matter junction, likely related to hypertension rather than CAA.

In an investigation of 20 surgically evacuated hematoma specimens from subjects with spontaneous ICH, 11 lobar and 9 from the basal ganglia, two microaneurysms were detected, one from the posterior parietal-occipital lobe related to CAA-associated angiitis and the other from the basal ganglia that also demonstrated fibrinoid necrosis [62]. In an autopsy study of 400 brains, CAA was seen in 91 subjects, three of whom demonstrated ICH, with hemorrhage attributed to CAA in one case. The patient was an 85-year-old male who had cerebellar hemorrhage in the setting of severe CAA, especially in vessels of the cerebellar cortex with four vessels showing aneurysmal dilatation, necrosis, and amyloid deposition around the site of hemorrhage. Eight subjects with CAA demonstrated fibrinoid necrosis without hemorrhage [63]. Extensive examination of an autopsy brain with severe CAA and CAA-related ICH utilizing computer-assisted three-dimensional reconstruction of vessels demonstrated microaneurysms arising from eight CAA-affected vessels in the superficial to mid cortex, occasionally with microhemorrhages in the vicinity [24]. Three of the eight microaneurysms demonstrated fibrinoid necrosis which appeared in thickened intima around the segment of maximum dilatation, some infiltrated by foamy macrophages [24]. Aβ deposits were absent or sparse in the most dilated portions of the microaneurysm but reappeared in the media and adventitia distal to the focus of maximum dilatation [24]. It has been shown in both familial and AD-associated CAA that vessels with severe fibrosis/hyalinization and microaneurysms often show sparse Aβ immunostaining but prominent macrophages [25]. Several CBAs in the setting of CAA in our cohort also demonstrated fibrosis and foamy macrophages accompanied by lack of Aβ staining in the aneurysmal portion while retaining immunoreactivity in the parent vessel (Fig. 1F). The extensive loss of the vessel wall with macrophage infiltration may underlie the lack of amyloid in the CBAs.

CBAs were predominantly seen in elderly individuals who had multiple cerebrovascular comorbidities, often severe. Many had infarcts with a few in the vicinity of CBAs, but only two microhemorrhages could be definitively associated with CBAs. At least in routine postmortem brain examination CBAs were rare, but they can be easily missed due to their small size and focality as many of the CBAs disappeared in deeper sections. Thus, the number of patients with CBAs is likely an underestimate due to sampling and assessment by different pathologists over time with our ability to confirm cases positive for CBAs but the unfeasibility of re-reviewing all negative cases. Additionally, CBAs are somewhat complex three-dimensional structures which may limit their accurate representation and analysis in two dimensional histologic sections. There is also a bias toward elderly patients with dementia, many of them with AD, as our subjects were part of routine hospital and dementia autopsies. Furthermore, the standard autopsy protocol examines fewer blocks from the brains of patients without dementia compared to patients with dementia that increase the likelihood of finding CBAs in subjects with dementia. The prevalence of CAA increases with age, and CAA is seen in the majority of patients with AD [8], which would explain the frequency of CAA in our cohort. However, the prevalence of CAA, especially mild CAA, may be underestimated in nondemented patients as immunohistochemistry for Aβ is not routinely performed in patients without a clinical history of cognitive impairment or without suspicion for CAA on H&E. They were also not selected for risk factors for ICH such as hypertension, prior stroke and/or hemorrhage although many had one or more of these comorbidities.

In conclusion, CBAs, rather than being a major source of ICH, may be one of the many manifestations of SVD encompassing both non-amyloid and amyloid vasculopathies which have been hypothesized to lie on a spectrum with common underlying mechanisms including BBB breakdown [29]. Loss of integrity of the BBB due to hypertension or CAA may lead to plasma protein insudation with fibrinoid necrosis and predispose to hemorrhage, while CBAs that undergo macrophage infiltration and fibrosis may have less proclivity for hemorrhage [25]. Many of the CBAs in our subjects were associated with CAA, and with the aging population and continued control of HTN, the proportion of both ICHs and CBAs associated with CAAs could increase. Although lowering blood pressure can decrease risk from hemorrhage in both hypertension and CAA-associated ICH [11], there is no current treatment or effective preventative measure for CAA. A better understanding of SVD and the mechanisms leading to ICH is needed for the development of preventative and therapeutic interventions against ICH.

## Data Availability

All data generated or analyzed during this study are included in this published article.
